# Antioxidant, Antimicrobial, and Metabolomic Characterization of Blanched Pomegranate Peel Extracts: Effect of Cultivar

**DOI:** 10.3390/molecules27092979

**Published:** 2022-05-06

**Authors:** Tandokazi Pamela Magangana, Nokwanda P. Makunga, Olaniyi Amos Fawole, Maria A. Stander, Umezuruike Linus Opara

**Affiliations:** 1 Department of Botany and Zoology, Stellenbosch University, Private Bag X1, Matieland, Stellenbosch 7602, South Africa; tkmagangana@sun.ac.za; 2 SARChI Postharvest Technology Research Laboratory, Faculty of AgriSciences, Africa Institute for Postharvest Technology, Stellenbosch University, Private Bag X1, Stellenbosch 7602, South Africa; 3 Postharvest Research Laboratory, Department of Botany and Plant Biotechnology, University of Johannesburg, P.O. Box 524, Auckland Park, Johannesburg 2006, South Africa; 4 Department of Biochemistry, Stellenbosch University, Private Bag X1, Matieland, Stellenbosch 7602, South Africa; lcms@sun.ac.za; 5 UNESCO International Centre for Biotechnology, Nsukka 410001, Nigeria

**Keywords:** antimicrobial activity, hot water blanching, cultivar variation, phytochemical content, pomegranate fruit peel, value addition

## Abstract

Hot water blanching at 80 °C for 3 min can be used as a novel pre-treatment step in pomegranate peel to preserve the integrity of the phytochemical content within the peel extracts by lowering or inactivating enzymes such as polyphenol (PPO) oxidase and peroxidase (POD) that are responsible for the break-down of phytochemicals within the peel. The aim of this study was to investigate the effect of hot water blanching pre-treatment on yield, bioactive compounds, antioxidants, enzyme inactivation, and antibacterial activity of ‘Wonderful’, ‘Acco’, and ‘Herskawitz’ pomegranate peel extracts. We used a variety of spectrophotometric-based assays and liquid chromatography mass spectrometry (LC-MS)-based approach to characterize and quantify metabolites within the peel extracts. Blanching significantly (*p* < 0.05) reduced PPO activity in all peel extracts, with the highest PPO reduction in ‘Herskawitz’ peel extracts at 0.25 U/mL. Furthermore, higher antioxidant activity in ‘Herskawitz’ blanched peel extracts using 2,2-diphenyl-1-picryl hydrazyl (DPPH) antioxidant activity, ferric ion reducing antioxidant power (FRAP), and 2,2-azino-bis(3-ethylbenzothiazoline-6-sulphonic acid (ABTS) radical scavenging activity at 567.78 ± 9.47 µmol Trolox/g DM, 800.05 ± 1.60 µmol Trolox/g DM, and 915.27 ± 0.61 µmol Trolox/g DM, respectively, was noted. ‘Herskawitz’ blanched peel extracts were recorded with the lowest minimum inhibitory concentration (MIC) value of 80 µg/mL for Gram-positive *Bacillus subtilis* and Gram-negative *Klebsiella pneumoniae* bacteria strains. A total of 30 metabolites were present in ‘Acco’ and ‘Herskawitz’ peel extracts and were tentatively identified after LC-MS profiling. This study demonstrates that blanched peel extracts from ‘Herskawitz’ cultivar have great potential for commercial use in value-added products in the nutraceutical, cosmeceutical, and pharmacological industries.

## 1. Introduction

Pomegranate (*Punica granatum* L.; Lythraceae) is a fruit-bearing deciduous tree or evergreen shrub extensively grown in many parts of the world, including South Africa [1]. The popularity and demand of the entire plant, its different anatomical parts, and the juice derived from the fruit are largely due to its versatile health-enhancing properties [2].

The fruit peel is considered a waste and is the largest part of the fruit, accounting for almost 49% to 55% of fruit weight, depending on the cultivar [3]. Of all the different plant parts of pomegranate, it possesses stronger antimicrobial [2,4], antioxidant [3,5], and anticancer activities [6,7], to name but a few. These biological activities are strongly linked to its chemical composition, especially the phenolics [8,9], as pomegranate peel is a good source of bioactive compounds including phenolic acids and flavonoids, ellagitannins, and many others [9,10,11,12,13].

On a global scale, there are more than a thousand pomegranate varieties available, with many more being constantly developed; however, ‘Wonderful’ remains the most popular cultivar in South Africa due to its exceptional properties and high yield [14]. It has the highest cultivar distribution of 76%, followed by ‘Acco’ and ‘Herskawitz’ each at 9% [15]. Several studies have reported the cultivar-dependent variability in bioactive compounds present in pomegranate peel [16,17,18,19]. For instance, Zhao et al. [20] reported that individual anthocyanin content differed according to the cultivar (‘Lvbaoshi‘, ‘Hongbaoshi’, and ‘Moshiliu’) and colouration stage, ‘Moshiliu’ was reported with the highest concentration of Cyanidin 3,5-diglucoside and delphinidin 3-glucoside at 53.52 mg/100 g and 34.36 mg/100 g, respectively. These findings were compared to ‘Lvbaoshi‘ and ‘Hongbaoshi’ pomegranate peel and were found to be higher by 34 times and 12 times for Cyanidin 3,5-diglucoside, and 68 times and 83 times for delphinidin 3-glucoside, respectively. Similarly, Saad et al. [21] noted the highest total phenolic content at 134.3 mg GAE/g DW in ‘Mekki‘ and lowest in ‘Jbeli’ at 181.0 mg GAE/g DW, while condensed tannins and hydrolyzable tannins were found in elevated concentrations in ‘Jbeli’ pomegranate peel at 3.1 mg C3GE/g DW and 504.8 mg TAE/g DW, respectively. Variations in tannin content between the different cultivars were attributed to genetic and environmental sampling differences. 

Variation of polyphenolics in different pomegranate peel cultivars may also be influenced by the processing factors used [22,23,24]. For instance, numerous drying methods may be capable of conserving the integrity of the phytochemical constituents found in pomegranate peel during processing [22,25,26]. However, this may be limited due to the availability and costs involved. Therefore, selecting the right processing method is essential in preserving the integrity of the product [22]. Pre-treatment protocols that are easy to set up and cost effective may result in a lower loss of valuable biological compounds and allow for the full exploitation of their most desirable health-promoting properties. Generally, hot water blanching is the most simple, easily accessible, and commercially accepted blanching technique; it involves the immersion of the product into hot water for temperatures of about 55 °C up to 100 °C for several minutes [27,28,29]. This process allows for the reduction or inactivation of enzymes such as peroxidase (POD) and polyphenol oxidase (PPO) that contribute to negative effects such as off-flavours, odours, undesirable colour, textures, and the loss of nutrients and phytochemicals [22,27,28,29]. The blanching pre-treatment technique has been criticized for greatly reducing phytochemicals and nutrients through leaching and large amounts of water waste after processing [27,28,30]. For instance, Hughey et al. [30] observed that almost 90% of polyphenols in almond skin leached into the water after blanching at 100 °C for 10 min. Likewise, Geertens et al. [31] reported alk(en)-ylresorcinol content losses in blanched mango peel ‘Nam Dokmai’ by up to 55%. Contrary to this report, Nurhuda et al. [28] reported a 39% increase of anthocyanin concentration in rambutan peel ‘Anak Sekolah’ after blanching at 100 °C for 2.5 min. Several other studies have reported blanching pre-treatment in fruit peel in which valuable bioactive compounds were preserved [29,32]. Differences in these findings are based on several factors such as the blanching method, heat sensitivity of various bioactive compounds and location within the plant structure, enzyme activity, plant material type, cultivar variations, and even ripeness [22,33]. The temperature and timing of blanching also play a significant role and should be suitably altered according to the type of fruit peel waste and sample size [22,33]. 

Although much has been published on the blanching of fruit peel waste [27,28,30,34], there are still little published data on the effects of hot water blanching on pomegranate peel waste. We previously reported that blanching at 80 °C for 3 min promotes higher antioxidant properties in ’Wonderful’ pomegranate peel extracts at three different harvest maturities [35]. To our knowledge, this is the first time that the effect of blanching on the quality attributes of three different cultivars, namely ‘Acco’, ‘Herskawitz’, and ‘Wonderful’ pomegranate peel extracts, has been studied. We thus hypothesized that blanching may reduce or inhibit PPO and POD enzyme activity, assisting in greater extractability of phytochemicals and resulting in stronger antibacterial activity. The effect of blanching may also differ due to cultivar differences. To test our hypothesis, an evaluation of phytochemical, antioxidant, and enzyme activity was conducted by a variety of spectrophotometric-based assays. Moreover, liquid chromatography-mass spectrometry (LC-MS) was used to assess the metabolic changes occurring in several compounds which are of commercial significance after the blanching process. Metabolomics using LC-MS has been utilized as a chemotaxonomic tool to provide insights into the plant’s diversity, evolution, and establishing cultivars with the highest metabolomic profiles [2,20,21]. This information can be used for quality control during product development of a value-added product and to improve the development of a natural antimicrobial drug [2,20]. 

This study also examined the effect of blanched peel extracts against *Klebsiella pneumoniae* ATCC 13883, *Escherichia coli* ATCC 11775, *Staphylococcus aureus* ATCC 12600, and *Bacillus subtilis* ATCC 6051 bacteria strains. Therefore, this study aimed to explore the possible effects of hot water blanching pre-treatment on the enzyme inactivation, antioxidant compounds, and antibacterial activity of ‘Wonderful’, ‘Acco’, and ‘Herskawitz’ pomegranate peel extracts.

## 2. Results

### 2.1. Polyphenol Analysis and Antioxidant Activity

The phenolic content of dried pomegranate peel was significantly (*p* < 0.05) higher in blanched peel compared to unblanched peel (Table 1). ‘Wonderful’ showed the highest extract yield at 33.83 ± 1.42% per g DM after blanching, with the lowest noted in ‘Herskawitz’ at 27.36 ± 0.60% per g DM. Moreover, blanched ‘Herskawitz’ peel extracts showed the highest TPC, TTC, TFC, and TAC at 15.74 ± 0.13 mg GAE/g DM, 1.92 ± 0.04 mg GAE/g DM, 1.70 ± 0.00 CE per g DM, and 0.15 ± 0.00 mg Cy3dE/g DM, respectively. The antioxidant activities of pomegranate peel extracts at different cultivars are shown in Table 1. Total antioxidant activity followed the same trend as phytochemical content, with blanched ‘Herskawitz’ peel extracts exhibiting the highest antioxidant activity for all three assays tested, namely DPPH, FRAP, and ABTS at 567.78 ± 9.47 µmol Trolox/g DM, 800.05 ± 1.60 µmol Trolox/g DM, and 915.27 ± 0.61 µmol Trolox/g DM, respectively. 

### 2.2. Polyphenol Oxidase (PPO) and Peroxidase (POD) Enzyme Activities

There was a significant (*p* < 0.05) decrease in PPO and POD activity for all blanched pomegranate peel extracts (Table 1). Polyphenol oxidase enzyme activity was lowest in blanched ‘Herskawitz’ and blanched ‘Acco’ at 0.25 ± 0.05 U/g FW and at 0.28 ± 0.03 U/g FW, respectively, which represents a 63% and 83% reduction in PPO activity, respectively. 

### 2.3. Antibacterial Activity

All ethanolic extracts investigated in this study showed good antibacterial activity, with the MIC values ranging between 80 µg/mL and 310 µmg/mL, regardless of bacterial strains tested (Table 2). Blanched pomegranate ‘Herskawitz’ peel extracts were recorded with the lowest MIC value of 80 µg /mL for Gram-positive (*Bacillus subtilis*) and Gram-negative (*Klebsiella pneumoniae*) strains. The same 80 µg/mL concentration was noted for blanched pomegranate ‘Acco’ peel extracts in only *Bacillus subtilis* bacteria strains. All blanched peel extracts had a two- or four-fold MIC value compared to unblanched peel (Table 2). 

### 2.4. Comparative Metabolomics of Cultivar

#### 2.4.1. Tentative Identification of Metabolites

**Figure 1:**
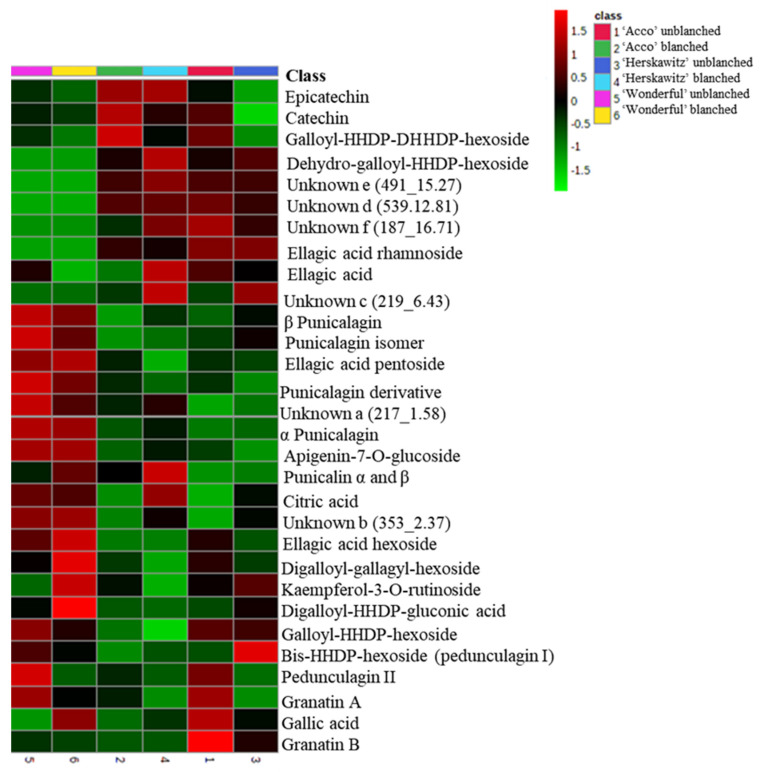
Heat map showing the relative abundance of the identified compounds between ‘Acco’, ‘Herskawitz’, and ‘Wonderful’pomegranate peel extracts. The red boxes show higher mean concentrations among different pomegranate fruit samples, while the green boxes show lower concentrations. All cultivars were harvested at the ripe stage (commercial harvest).

The largest peaks, presented in Figure S1, were used to characterize the profiles of the major chemicals present in ‘Wonderful‘, ‘Acco’, and ‘Herskawitz’ pomegranate peel extracts (Table 3). A total of 30 chemicals, including phenolic acids (**5**), organic acids (**1**), flavonoids (**4**), ellagitannins (**14**), and other unknown polyphenols (**6**), were present in ‘Acco’ and ‘Herskawitz’ pomegranate peel extracts and were tentatively identified after LC-MS profiling. Of the 30 chemicals, only 25 metabolites were present in ‘Wonderful‘ peel extracts, with 5 chemicals absent, namely ellagic acid rhamnoside, dehydro-galloyl-hexahydroxydiphenol-hexoside, and unknowns c, d, and f. The relative abundance of the identified chemicals for each cultivar are shown in the form of a heat map (Figure 1), where some metabolites are found in high concentrations (denoted by red colour) and low concentrations (denoted by green colour).

#### 2.4.2. Chemotype Differences

**Figure 2:**
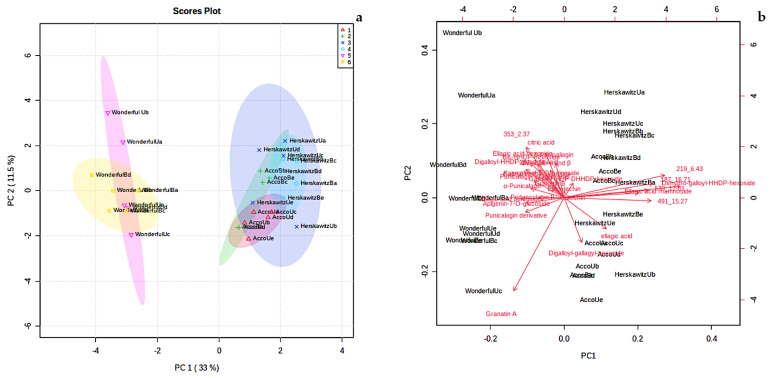
(**a**) Principal component analysis (PCA) score plot showing variation of *P. granatum* L. fruit peel from three different cultivars blanched at 80 °C for 3 min, i.e., ‘Acco’ unblanched (red colour), ‘Acco’ blanched (green colour), ‘Herskawitz’ unblanched (blue colour), ‘Herskawitz’ blanched (cyan colour), ‘Wonderful’ unblanched (purple colour), and ‘Wonderful’ blanched (yellow colour) peel extracts; (**b**) Principal component analysis (PCA) biplot showing the compounds that influenced the differentiation of collections into distinct clusters.

The level of variation associated with principal component (PC) 1 was 33%, whereas principal component 2 explained 11.5% of the variation (Figure 2a). Principal component 1 usually accounts for the major variation on the x-axis, while PC 2 accounts for the minor variation on the y-axis. The score plot showed six detectable groupings, which include ‘Acco’ unblanched (red colour), ‘Acco’ blanched (green colour), ‘Herskawitz’ unblanched (blue colour), ‘Herskawitz’ blanched (cyan colour), ‘Wonderful’ unblanched (purple colour), and ‘Wonderful’ blanched (yellow colour) peel extracts (Figure 2a). A biplot was generated to identify the features responsible for the clustering patterns illustrated in the score plot (Figure 2b). Eight metabolites were identified as the discriminants of the six groupings of *P. granatum* L., namely, bis-HHDP-hexoside (pedunculagin I), dehydro-galloyl-HHDP-hexoside, ellagic acid rhamnoside, and unknown b, c, d, e, and f (with [M-H]- at *m*/*z* 353.0731, 219.0497, 539.216, 491.0756, and 187.1022, respectively, and retention times of 2.37 min, 6.43 min, 12.81 min, 15.27 min, and 16.71 min, respectively). Most of these compounds belong to the ellagitannins group and unknown metabolites (refer to Table 3).

**Figure 3:**
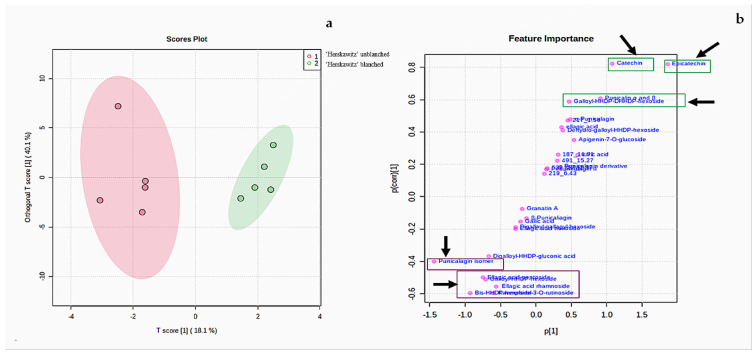
(**a**) Orthogonal partial least squares discriminant analysis (OPLS-DA) for ‘Herskawitz’ unblanched (purple colour) and ‘Herskawitz’ blanched (green colour) peel samples. (**b**) S-plot of orthogonal partial least squares discriminant analysis of LC-MS spectra for ‘Herskawitz’ unblanched and ‘Herskawitz’ blanched peel samples showing the compounds that are important discriminants.

According to the two PC scores in Figure 2a, ‘Herskawitz’ unblanched (blue colour) and ‘Herskawitz’ blanched (cyan colour) were chemically more similar and appeared to cluster closer to each other in their sub-group on the PCA. This led to the use of the orthogonal partial least squares discriminant analysis (OPLS-DA) test to further distinguish between the two groups (Figure 3a). From this, it was apparent that ‘Herskawitz’ unblanched (purple colour) and ‘Herskawitz’ blanched (green colour) were distinct from each other based on their chemical composition (Figure 3a). The S-plot shown in Figure 3b was used to visualize the spectral features that contributed to the metabolite variation in ‘Herskawitz’ peel extracts. The chemical discriminants identified in ‘Herskawitz’ unblanched were punicalagin isomer, bis-HHDP-hexoside (pedunculagin I), ellagic acid rhamnoside, ellagic acid pentoside, granatin B, and galloyl-HHDP hexoside, whereas those from ‘Herskawitz’ blanched were catechin, epicatechin, galloyl-HHDP-DHHDP-hexoside, and punicalin α and β.

**Figure 4:**
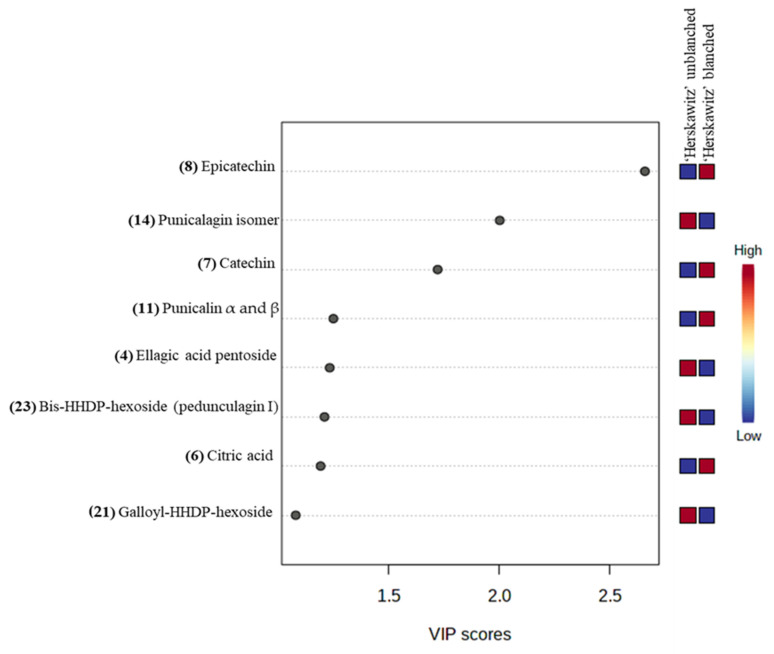
Variable importance in the projection (VIP) plot showing the eight most statistically significant metabolites linked to the changes in ‘Herskawitz’ unblanched and ‘Herskawitz’ blanched peel extracts.

For further analysis of the significant metabolites, a “variable importance in projection” (VIP) score was constructed (Figure 4). The value of a VIP score that is equal to or greater than 1 (VIP score ≥ 1) is the typical rule for selecting relevant variables or statistically important metabolites [40]. The VIP score in Figure 4 showed that ‘Herskawitz’ blanched peel extracts had the highest relative abundance of epicatechin, catechin, punicalin α and β, and citric acid, while punicalagin isomer, ellagic acid pentoside, bis-HHDP-hexoside (pedunculagin I), and galloyl-HHDP-hexoside were down-regulated as a result of blanching. The VIP plot, score plot, and biplot for the other two cultivars with their treatments (‘Acco’ unblanched, ‘Acco’ blanched, ‘Wonderful’ unblanched, and ‘Wonderful’ unblanched peel samples) are included in the Supplementary Data (Figures S3 and S4). 

**Figure 5:**
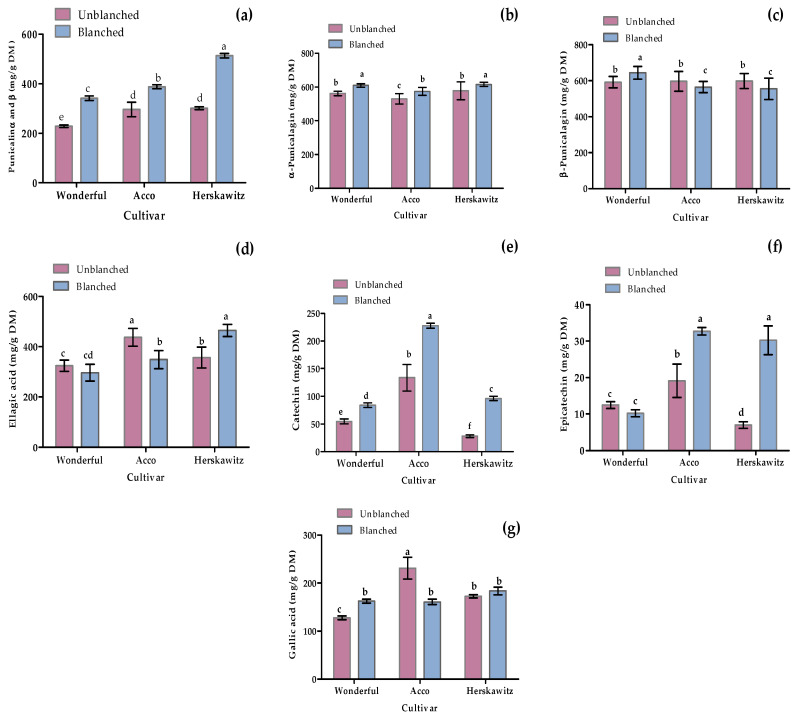
Individual phenolic and flavonoid concentration from three different cultivars, namely ‘Wonderful’, ‘Acco’, and ‘Herskawitz’ pomegranate peel at ripe harvest maturity blanched at 80 °C for 3 min. Graphs represent concentrations in mg/g DM for (**a**) punicalin α and β; (**b**) α-punicalagin; (**c**) β-punicalagin; (**d**) ellagic acid; (**e**) catechin; (**f**) Epicatechin; and (**g**) gallic acid. Bars with different letter(s) represent statistical differences (*p* < 0.05) using Duncan’s multiple range test.

As shown in Figure 5, seven polyphenols were targeted to quantify through LC-MS profiling, including two phenolic acids (ellagic acid and gallic acid), two flavonoids (catechin and epicatechin), and three (punicalagin α, punicalagin β, and punicalin α and β) based on the previously reported antioxidant and antibacterial activities [2,3,8]. Blanching had a significant (*p* < 0.05) positive effect in promoting the up-regulation of certain metabolites, such as punicalin α and β, α-punicalagin, catechin, and epicatechin, for all three cultivars tested. Moreover, ‘Herskawitz’ blanched peel samples were reported with the highest punicalin α and β, α-punicalagin, epicatechin, and ellagic acid concentration at 513 ± 9.09 mg/g DM, 615 ± 12.49 mg/g DM, 30.95 ± 3.95 mg/g DM, and 464.85 ± 24.05 mg/g DM, respectively.

### 2.5. Correlation Matrix

Pearson’s correlation was used to determine the degree of correlation between selected reference data and variables (Table 4). As expected, TPC was positively correlated with TTC, TAC, FRAP, DPPH, and ABTS at *r* = 0.984, *p* = 0.05; *r* = 0.879, *p* = 0.05; *r* = 0.969, *p* = 0.05; *r* = 0.992, *p* = 0.05; and *r* = 0.895, *p* = 0.05, respectively. In addition, strong positive correlations between the antioxidant assays were reported at *r* = 0.969, *p* = 0.05, between DPPH and FRAP; at *r* = 0.842, *p* = 0.05, between FRAP and ABTS; and at *r* = 0.891, *p* = 0.05, between DPPH and ABTS. This is in agreement with our results in Table 1 that show that high phenolic content in peel extracts is a direct cause of the strong antioxidant activity. Moreover, the Pearson correlation matrix showed a significant (*p* < 0.05) high negative relationship between POD enzyme activity and extract yield at *r* = −0.982, suggesting that the decrease in POD enzyme activity as a result of blanching contributes to higher extract yield antioxidant activity (Table 4). Strong negative correlations were reported between FRAP and *B. subtilis* (*r* = −0.854, *p* < 0.05), DPPH and *B. subtilis* (*r* = −0.910, *p* < 0.05), TTC and *B. subtilis* (*r*= −0.916, *p* < 0.05), TPC and *B. subtilis* (*r* = −0.942, *p* < 0.05), *B. subtilis* and ABTS (*r* = −0.856, *p* < 0.05), *B. subtilis* and punicalin α and β ( *r*= −0.835, *p* < 0.05), ABTS and *K. pneumoniae* (*r* = −0.889, *p* < 0.05), punicalin α and β and *K. pneumoniae* (*r* = −0.838, *p* < 0.05), *K. pneumoniae* and TFC (*r* = −0.820, *p* < 0.05), and *K. pneumoniae* and β punicalagin (*r* = −0.861, *p* < 0.05) (Table 4).

## 3. Discussion

For all three cultivars, the ‘Herskawitz’ peel extracts exhibited the highest phenolic content and antioxidant properties, while ‘Wonderful’ peel extracts exhibited the lowest (Table 1). Furthermore, a total of 30 major chemicals in ‘Acco’ and ‘Herskawitz’ peel extracts were tentatively identified after LC-MS profiling, while ‘Wonderful‘ peel extracts only had a total of 25 metabolites (Table 3). Inter-genetic cultivar variability may likely be responsible for the change noted in the chemical composition and biochemical features [2,16,20]. In addition, possible changes in chemical composition may also be related to the plants’ adaptation to the changing environments as a response to global climate change [41]. For example, Nasrabadi et al. [42] studied the influence of water stress on the biochemical traits of two Iranian pomegranate cultivars, namely, ‘Malas Yazdi’ and ‘Shishecap’. They noted that the total phenolic content (TPC) and antioxidant activity significantly increased with an increase in water stress in ‘Shishecap’ cultivar (TPC: 450 to 490 mg GAE g^−1^ FW; 58% to 70% antioxidant activity), while no significant changes in ‘Malas Yazdi’ were reported. They attributed this to the cultivar origin of ‘Malas Yazdi’, as it originated from Yazd province, a desert zone. Moreover, understanding the effect of temperature and water availability on plants may help to explain or determine the degree of acclimatization [41]. Such genetic variability in the past has been exploited within crop species to meet subsistence food requirements, especially for the improvement of phenolic yields [43]. For instance, Adiba et al. [44] assessed 11 Mediterranean pomegranate cultivars (Zheri Automne, Bzou, Djebali, Gjeibi, Gordo de Jativa, Zheri Precoce, Sefri, Grenade Rouge, Mollar Osin Hueso, Grenade Jaune, and Ounk Hmam) for drought tolerance based on their responses to severe water stress. They noted clear differences among the pomegranate cultivars in response to water stress, with the most frequent effects of water stress recorded on yield, fruit weight, and chlorophyll pigment contents. Among the 11 pomegranate cultivars studied, the fruit weight of Ounk Hmam cultivar was significantly reduced by 51%, while fruit weight reduction in other cultivars was significantly lower by 14% and Zheri Automne cultivar was not affected. 

All peel extracts, regardless of cultivar variation and blanching effect, had high phenolic content and antioxidant activities. This is likely the cause of ultrasound-assisted extraction with 70% ethanol at temperature 40 ± 5 °C, extraction time of 1 h, solvent ratio (15:1 *w*/*w* dry weight; solvent/sample), and maximum power and extraction frequency of 700 W and 40 kHz, respectively, previously reported by Magangana et al. [45]. These conditions were noted to improve the penetration rate of solvent into the plant cellulosic material of ‘Wonderful’ pomegranate peel extracts by, possibly, breaking down the plant cell wall and, thus, leading to the release of bound phenolics during extraction. For instance, Liu et al. [46] evaluated the pomegranate peel-derived punicalagin extracted using ultrasonic-assisted extraction. They obtained the highest punicalagin at 505 ± 1.73 mg/g DW under the following conditions: extraction time of 25 min, temperature of 25 °C, 53% ethanol, sample-to-liquid ratio of 1:25 *w*/*v*, and ultrasonic power of 757 W. Several authors have reported similar findings on pomegranate peel extracts [22,47,48]. The ultrasonic extraction method could be used by those in the industry that have access to the instrument.

Hot water blanching of pomegranate peel significantly (*p* < 0.05) affected the phenolic content (extract yield, TPC, TTC, TFC, and TAC) in all of the cultivars, with higher phenolic content in blanched peel extracts compared to the unblanched peel extracts (Table 1). For instance, the highest extract yield was recorded in blanched ‘Wonderful’ peel extracts and least in blanched ‘Herskawitz’ peel extracts (Table 1). This is particularly interesting as extract yield is considered one of the most important indicators of an effective extraction process for producing high-quality products [49]. In our study, the extract yield results were higher than those reported by Bustamante et al. [50], ranging from 0.2 to 8.5% (*w*/*w*) for ‘Wonderful’ peel extracts using supercritical carbon dioxide extraction. Similarly, in another study by Sharayei et al. [51], the extract yield for ’Sishe Kape-Ferdos’ peel extracts was noted at 13.1% (*w*/*w*) using ultrasound-assisted extraction. Malviya et al. [52] also reported the highest extract yield at 16.3% (*w*/*w*) from ‘Ganesh’ peel extracts using 50% ethanol solvent. Interestingly, the highest phenolic content (TPC, TTC, TFC, and TAC) was found in blanched ‘Herskawitz’ peel extracts and the least in blanched ‘Wonderful’ peel extracts regardless of the high extract yield reported in blanched ‘Wonderful’ peel extract (Table 1). The variation in phenolic content between the blanched and unblanched peel suggests a level of sensitivity of TPC, TTC, TFC, and TAC to temperature treatment. These results suggest that blanching at optimum temperature and time combination caused a significant (*p* < 0.05) increase in phenolic content in all of the studied cultivars, especially blanched ‘Herskawitz’ peel extracts. Optimum temperature and time combinations during blanching result in cellulosic disruptions that cause significant phytochemical release from the plant compartment (vacuole), subsequently resulting in increased phytochemical extractability [22,28,33]. The differences in extract yield and other phenolic content are likely be due to heat sensitivity during blanching of the various cultivars studied. 

Both qualitative and quantitative differences in the different peel extracts are strongly influenced by cultivar differences and blanching treatments. For instance, there was up-regulation of numerous chemicals in blanched ‘Herskawitz’ peel extracts (Figure 1, Figure 2 and Figure 3). Furthermore, higher metabolite concentrations for punicalin α and β, α-punicalagin, epicatechin, and ellagic acid at 513 ± 9.09 mg/g DM, 615 ± 12.49 mg/g DM, 30.95 ± 3.95 mg/g DM, and 464.85 ± 24.05 mg/g DM, respectively, were noted (Figure 5). This data could explain the higher phenolic content and antioxidant activity reported in blanched peel extracts, especially Herskawitz’ peel extracts (Table 1). It could also explain the strong antibacterial activity in all blanched peel extracts with MIC values ranging between 80 µg/mL and 310 µg/mL, regardless of bacterial strains tested, which was particularly stronger for blanched ‘Herskawitz’ and blanched ‘Acco’ peel extracts with the lowest MIC value of 80 µg/mL for both Gram-positive *B. subtilis* and Gram-negative *K*. *pneumoniae* bacteria strains (Table 2). Differences in the antibacterial activities in the peel extracts may be linked to variations in phenolic content of extracts and strains sensitivity [2,22,53,54,55]. It is thought that biological activity, including antimicrobial properties of pomegranate peel, results from the combined effect of polyphenols such as hydrolyzable tannins (punicalagin) and other bioactive compounds and not just one compound, suggesting additive and synergistic interactions of a complex mixture of phytochemicals [56,57,58]. Additionally, Pearson’s correlation heatmap in cluster 3 consisting of blanched ‘Herskawitz’ and blanched ‘Acco’ samples showed higher expression of all bioactives tested (Figure S2, Table 4). As expected, strong negative correlations were reported between phenolic content in the form of TPC, TTC, TFC, punicalin α and β, and β punicalagin, as well as with antioxidant activity in the form of FRAP, DPPH, and ABTS with *B. subtilis* and *K*. *pneumoniae* (Table 4, Figure S2). This suggests that a higher recovery of phenolic content in the form of TPC, TTC, TFC, punicalin α and β, and β punicalagin may lead to improved antioxidant activities (DPPH, ABTS, and FRAP), which is responsible for the strong antibacterial activities shown by all peel extracts, especially those produced by blanched ‘Herskawitz’ peel and blanched ‘Acco’ peel for both *B. subtilis* and *K. pneumoniae* (Table 2).

Similarly, strong positive correlations between punicalin α and β and TTC at *r* = 0.885, *p* < 0.05; punicalin α and β and TPC at *r* = 0.824, *p* < 0.05; TAC and punicalin α and β at *r* = 0.930, *p* < 0.05; and β punicalagin and TFC at *r* = 0.955, *p* < 0.05, were recorded. In contrast, strong negative correlations between β punicalagin and PPO enzyme activity were also noted at *r* = −0.828, *p* < 0.05, suggesting that lowered activity of PPO enzyme during blanching treatment may be the direct cause for the improved extraction of β punicalagin in pomegranate peel extracts (Table 4). Thermal stability of different polyphenols is a key factor determining the degradation rate during blanching [30,59]. Moreover, the response to heat stress of a plant depends on various factors such as the cultivar type, harvest maturity, and intensity and period of exposure to the stress [60]. Some plants respond by inactivating enzymes such as PPO and POD responsible for the degradation of phytochemical content within the plant source which allows for a higher extraction of valuable phytochemicals [22,27,28]. We reported low PPO and POD in all three cultivars after blanching (Table 1). Polyphenol oxidase enzyme activity after blanching was the lowest in ‘Herskawitz’ peel extracts at 0.25 ± 0.05 U/g FW, followed by ‘Acco’ at 0.28 ± 0.03 U/g FW and ‘Wonderful’ peel extracts at 0.33 ± 0.03 U/g FW. These data lend support to the higher phenolic concentrations and antioxidant activities reported in ‘Herskawitz’ blanched peel samples due to lowered POD and PPO enzyme activity during blanching (Table 1). Contrary to our findings, a reduction of TPC in yellow passion fruit peel [61] and yam peel [62] were reported due to the leaching of water-soluble polyphenols from the peel during blanching. Moreover, the extent to which heat-sensitive phytochemicals degrade during blanching may also be different even within the same plant source [59]. Temperature and time combinations affect the amount of polyphenols that leach into the water during blanching. Hughey et al. [30] noted that the degradation rate of polyphenols in almond skin was significantly higher, with almost 90% of polyphenols leaching into the water after blanching at 100 °C for 10 min compared to 30% loss of polyphenols at 25 °C for 10 min. Similarly, Geertens et al. [31] noted reductions of alk(en)-ylresorcinol contents in blanched ‘Tommy Atkins’ mango peel by 19% and 9% for unripe and ripe peel and in ’Nam Dokmai’ by 39% and 55%, respectively, after blanching. These losses of polyphenols were credited to both thermal degradation and leaching. Similar to our findings, Heras-Ramírez et al. [63] reported that blanching treatment significantly (*p* < 0.05) increased the retention of TFC in apple pomace, and attributed the TFC increase to PPO deactivation during blanching, which led to an increased stability of flavonoids during drying. Mai et al. [34] investigated the effects of blanching temperature (70, 80, 90, and 98 ± 2 °C), at a constant blanching time of 180 s, on the PPO and POD activities as well as the stability of betacyanin, phenolics, and antioxidant activity in pitaya peel. They noted significant reductions in PPO and POD activities as well as lowered antioxidant content with an increase in blanching temperature. Moreover, during storage of the blanched pitaya peel and unblanched peel, the loss of betacyanins and phenolics of blanched pitaya peel were 1.4 and 1.8 times lower than those of the unblanched peel, respectively. Numerous authors have reported an improved recovery of phenolic and antioxidant content after hot water blanching in fruit and vegetable peel, as well as pomegranate seeds and arils [1,28,29,32,64,65,66]. The blanching technique is well recognized as an important and inexpensive method capable of preserving valuable secondary metabolites during processing. Moreover, this particular study has shown that blanching at 80 °C for 3 min decreases enzymatic activity of PPO and POD that are responsible for the degradation of valuable secondary metabolites found in the pomegranate peel extracts of all three cultivars such as ‘Acco’, ‘Herskawitz’, and ‘Wonderful’ studied, and assists in greater extraction of phytochemicals within the peel extracts. It is evident that blanching at 80 °C for 3 min could aid in the recovery and preservation of valuable polyphenols that are useful for value addition in various industries on a commercial scale and should be considered as a novel, simple pre-treatment process in the pomegranate industry.

## 4. Materials and Methods

### 4.1. Plant Collection and Blanching Pre-Treatment

Three pomegranate cultivars (‘Acco’, ‘Herskawitz’, and ‘Wonderful’) from a commercial pomegranate orchard in Blydeverwacht farm, Wellington (33°48′0″ S, 19°53′0″ E), in the Western Cape Province, South Africa, were investigated. These cultivars were classified as sweet, sour, and sweet–sour, respectively, according to Fawole et al. [67]. The orchard is located on sandy loam soil, and the trees received the same fertilizer program and irrigation, delivering about 32 L ha^−1^. Day^−1^. The trees used were at a planting distance of 5 m × 3 m, with the same row orientation and tree training; 10 trees per cultivar were randomly selected for harvesting. A sample of 20 healthy fruit per cultivar of the same size and without defect was randomly collected manually from different positions of 10 randomly selected healthy adult trees between 5 and 7 years, with drip irrigation on 28 February, 15 March, and 29 March 2019, respectively. All cultivars shared the same environmental, cultivation, and soil conditions. The fruit were then transported to the Postharvest Technology Research Laboratory at Stellenbosch University in an air-conditioned car and stored at 7.5 ± 0.5 °C and 92 ± 3% relative humidity (RH) prior to processing for quality preservation using the method of Magangana et al. [45].

Fruit peels were separated from the edible arils, with a knife used to cut the peel into small pieces of proportions of approximately 20 ± 0.5 mm for both length and width and thickness of 5 ± 0.5 mm [3]. The initial moisture content for 10 g of pomegranate peel (per cultivar) was determined using a moisture analyzer (KERN DBS 60-3 Balingen, Germany) at 100 °C [1]. This was performed in triplicate for each cultivar and an average was reported. The initial moisture content for the fresh peel for cultivars ‘Acco’, ‘Herskawitz’, and ‘Wonderful’ was 69.29%, 74.51%, and 76.62% (*w*/*w*), respectively. Blanching pre-treatment was performed according to Magangana et al. [45]; briefly, the freshly cut pomegranate peel slices (100 g) were immersed in a water bath (Scientific, Maraisburg, South Africa) at 80 ± 2 °C for 3 min. Afterwards, blanched peels were soaked in iced water at 0 °C for 30 s and drained before weighing. This blanching pre-treatment process was repeated three times for each cultivar. Unblanched peels were used as the control, with the experiment performed in triplicate.

### 4.2. Peel Drying Procedure

After blanching, peels (100 g) were weighed using a digital balance (ML3002.E, Mettler Toledo, Switzerland) prior to drying in the oven (Model nr.072160, Prolab Instruments, Sep Sci., South Africa) operated at 60 ± 2 °C, at a relative humidity of 18.63% and 1.0 m/s air velocity. This was continued until the desired moisture content of 8% (*w*/*w*) was achieved [49] with slight modifications. For the enzyme assays, blanched peels were not dried (refer to Section 4.6). 

### 4.3. Ultrasound-Assisted Solvent Extraction

For all of the cultivars, ultrasound-assisted solvent extraction was used to extract phytochemicals from ground pomegranate peel of less than 1 mm, following the method described by Wang et al. [49], with slight modifications described by Magangana et al. [45]. Briefly, an ultrasonic bath (500 mm × 300 mm× 150 mm; Scientific, Cape Town, South Africa) operated at 700 W, frequency: 40 kHz and internal dimensions of) was used for peel extraction. The ultrasound extraction was carried out using 70% ethanol (on a solid–solid ratio of 15:1 (*w*/*v*), temperature of 40 ± 5 °C, and extraction time of 1 h). Extractions were conducted in triplicate for downstream experiments. 

### 4.4. Polyphenol Analysis 

#### Determination of Extract Yield, Total Phenolic Content, Total Tannin Content, Total Flavonoid Content, and Total Anthocyanin Content 

After drying the liquid extracts subjected to ultrasound-assisted solvent extraction (for details, refer to Section 4.3), dried peel samples were then weighed in a digital balance (ML3002, Mettler Toledo, Switzerland) to calculate extract yield. Equation (1) was used for yield calculation:

(1) Total extract yield (%)=g dried extract/100 g peel powder ×100 

The extract yield is expressed as % per g DM.

Total phenolic content (TPC) was determined according to the Folin–Ciocalteu colorimetric method described by Fawole et al. [2], with slight changes as stipulated in the work of Magangana et al. [45]. The test was carried out in triplicate. Gallic acid was used as a standard for the calibration curve (0–0.014 mg/mL). The total phenolic content was expressed as mg gallic acid equivalents (GAE) per g dry mass. The determination of total tannin content (TTC) was performed spectrometrically according to the procedure described by Makkar [68], with slight modifications by Magangana et al. [45]. Briefly, peel extracts (1 mL) were mixed with 1 mL of distilled water and 100 mg of polyvinylpolypyrrolidone (PVPP). This mixture was shaken thoroughly using a vortex for 30 s and incubated for 15 min in the dark at 4 °C. The mixture was then subjected to centrifugation at 4000× *g* for 10 min. Then, 50 µL of the supernatant was dissolved in 50% methanol, followed by adding 500 µL of Folin–Ciocalteu colorimetric reagent and, after 2 min, 2.5 mL of 2% sodium carbonate. The mixture was then thoroughly mixed by using a vortex for 30 s, followed by incubation at room temperature in the dark for 40 min. The absorbance was later taken after incubation at 725 nm using a UV-visible spectrophotometer (Thermo Scientific Technologies, Madison, WI, USA). Peel extracts not treated with PVPP were measured for total phenolic content, with equation (2) was used for TTC calculation:

(2) $$\mathrm{TTC}={\mathrm{TPC}}_{\left(\mathrm{in}\;\mathrm{peel}\;\mathrm{extract}\;\mathrm{without}\;\mathrm{PVPP}\right)}-{\mathrm{TPC}}_{\left(\mathrm{in}\;\mathrm{peel}\;\mathrm{extract}\;\mathrm{treated}\;\mathrm{with}\;\mathrm{PVPP}\right)}\;$$

Results are recorded as mg gallic acid equivalent per g peel extract (mg GAE/g DM).

The total flavonoid content (TFC) of the extracts was measured according to the colorimetric assay of Yang et al. [69], with slight modifications by Magangana et al. [45]. Ethanolic peel extract (1:10 *w*/*v*) was added to 300 µL sodium nitrite solution (5%) followed by 300 µL aluminium chloride (10%). The mixtures were incubated at room temperature for 5 min and then 2 mL of 1 mol/L sodium hydroxide was added. Immediately, the volume of the reaction mixture was made to 10 mL with distilled water and then thoroughly vortexed for 30 s. The absorbance of the mixture was determined at 510 nm using a spectrophotometer (Thermo Scientific Technologies, Madison, WI, USA). The calibration curve was prepared from different concentrations of catechin, with the calibration curve range 0–0.5 µg/mL. Triplicate measurements were taken for all samples. The flavonoid content was reported as mg catechin equivalents (CE) per g dry mass. The total anthocyanin content of the extracts was measured using the protocol of Wrolstad [70]. Briefly, 1 mL of the peel extract was mixed with 9 mL of pH buffers of 1.0 and 4.5, respectively. The absorbance was then taken using a spectrophotometer (Thermo Scientific Technologies, Madison, WI, USA) at 520 nm and 700 nm for each of the two buffers, respectively. Triplicate measurements were taken for each sample. The results are recorded as Cyanidin 3-glucoside equivalent (C3GE) per g dry matter (mg C3GE/g DM), with Equations (3) and (4) used to calculate the total absorbance and total anthocyanin content, respectively. The final results are reported as:

(3) $$A={\left(A510-A700\right)}_{\mathrm{pH}1.0}-{\left(A510-A700\right)}_{\mathrm{pH}4.5}$$

(4) $$\mathrm{Total}\;\mathrm{anthocyanin}\;\left(µg/\mathrm{mL}\right)=\frac{\left(A\times \mathrm{MW}\;\times \;\mathrm{DF}\right)}{\varepsilon \;\times \;L}$$

*A* = Absorbance, ε *=* Cyanidin-3-glucoside molar absorbance (26,900), MW = anthocyanin molecular weight of 449.2, DF = dilution factor, L = cell path-length (1.0 cm).

### 4.5. Antioxidant Activity 

#### Determination of Radical Scavenging Activity (RSA), Ferric Ion Reducing Antioxidant Power (FRAP), and 2,2-Azino-bis(3-ethylbenzothiazoline-6-sulphonic Acid (ABTS) Antioxidant Assay 

The method described by Karioti et al. [71], with slight modifications as described by Magangana et al. [45], was used to determine the DPPH radical scavenging capacity of pomegranate peel extracts. A 0.1 mM solution of DPPH (750 µM) in methanol was prepared and pomegranate peel extract (15 µL) was mixed with 735 µL methanol. This mixture was vortexed before incubation in the dark for 30 min. The absorbance was measured using a UV-visible spectrophotometer (Thermo Scientific Technologies, Madison, WI, USA) at 517 nm. Each sample was tested in triplicate, and sample quantification was based on the standard curve of 0–1500 μM. Results were expressed as µmol Trolox/g DM. The ferric ion reducing antioxidant power (FRAP) assay was used according to a method described by Benzie and Strain [72], with slight modifications by Magangana et al. [45]. Peel extracts (1 mL) were mixed with 10 mL of 50% methanol, sonicated in cold water, and then centrifuged at 4000× *g* (at 4 °C) for 5 min. Aliquots of 150 µL sample extract were added to 2850 µL of freshly prepared FRAP reagent consisting of 25 mL of acetate buffer (300 mM, pH 3.6), 2.5 mL of 2,4,6-Tris [2-pyridyl]-s-triazine (TPTZ) solution (10 mM TPTZ in 40 mM HCl), and 2.5 mL of FeCl_3_) solution (20 mM FeCl_3_·6H_2_O in water). The mixture was vortexed for 30 s and later incubated in the dark at room temperature for 30 min. The absorbance was taken at 593 nm using a UV-vis spectrophotometer (Thermo Scientific Technologies, Madison, WI, USA). Each sample was tested in triplicate, with the results expressed as µmol Trolox/g DM. The ABTS radical cation (ABTS^+^) stock solution was prepared according to Chirinos et al. [73], with minor modifications as stipulated in the work of Magangana et al. [45]. Briefly, ABTS stock solution was prepared by mixing equal volumes of ABTS solution (7.4 mM) and potassium persulfate solution (2.6 mM) and kept in the dark at 25 °C for 12 h. The absorbance was adjusted to 0.70 ± 0.02 after the incubation period using 80% (*v*/*v*) methanol. A 15 µL peel extract was added to 200 µL of the ABTS^+^ working solution, followed by incubation in the dark for 6 min. The absorbance was recorded at 750 nm using a microplate reader (Thermo Fisher Scientific, Shanghai, China). The results were expressed as µmol Trolox/g DM. Measurement of the antioxidant activity of different peel extracts was performed in triplicate.

### 4.6. Enzyme Activity

#### Determination of Polyphenol Oxidase and Peroxidase Enzyme

Preparation of samples for polyphenol oxidase and peroxidase enzyme extraction were determined according to the method of Magangana et al. [45]. Briefly, to further evaluate the influence of blanching on PPO and POD enzyme activity, 1 g of blanched peel, without drying, was ground into a fine powder using a pestle and mortar with liquid nitrogen. After that, 1 g of the powder was used for extraction with the addition of 10 mL of cold extraction buffer containing 0.1 M phosphate buffer at pH 7, 0.05 M/L EDTA, and 60 g/L polyvinyl polypyrrolidone at a ratio of 1:1:1, respectively. The samples were then vortexed for 30 s, followed by sonication in an ultrasonic bath (Scientific, South Africa) for 10 min and kept in the dark for 2 h at 4 °C. The mixture was then subjected to centrifugation at 4000× *g* rpm for 25 min at 4 °C, before the supernatant was carefully collected into clean vials and used as crude enzyme extract.

The activity of PPO enzymes was measured using a method by Gonzalez et al. [74], with minor modifications by Magangana et al. [45]. In this study, catechol was used as the substrate. Briefly, 2.5 mL of potassium phosphate buffer (0.1 M, pH 6) and 0.3 mL (0.1 M) catechol solution were added to a 3 mL reaction mixture. An enzyme extract (0.2 mL) was then added to the reaction mixture to initiate the reaction. The change in absorbance was monitored at a wavelength of 420 nm using a UV-vis spectrophotometer (Thermo Scientific Technologies, Madison, WI, USA) over a 3 min period at 25 °C. The blank cuvette contained extraction solution without the enzyme extract, with the results expressed as a unit per g of fresh weight (U/g FW).

Peroxidase enzyme activity was carried out using a spectrophotometric method employing a UV-vis spectrophotometer (Thermo Scientific Technologies, Madison, WI, USA) at 470 nm absorbance. In this study, guaiacol and hydrogen peroxide were used as substrates as described by Meighani et al. [75], with minor modifications by Magangana et al. [45]. Briefly, 2.73 mL of sodium phosphate buffer (0.1 M, pH 6), 0.1 mL of guaiacol (0.045 M), and 0.15 mL of hydrogen peroxide solution were added to a 3 mL cuvette. Subsequently, 0.02 mL of the enzyme extract was added to initiate the reaction, with the change in absorbance recorded at 470 nm over a 2 min period. The blank cuvette contained extraction solution without the enzyme extract, with the results reported as a unit per g fresh weight (U/g FW).

### 4.7. Ultra-Performance Liquid Chromatography Mass Spectrometry

Analyses were carried out on a Waters Synapt G2 quadrupole time-of-flight mass spectrometer (Milford, MA, USA) according to the method by Magangana et al. [45]. Briefly, the instrument consisted of a Waters Acquity ultra-performance liquid chromatograph (UPLC) coupled to an Acquity photo diode array (PDA) detector and operated in negative ion mode for electrospray ionization (ESI). The capillary voltage, cone voltage, desolvation temperature, and desolvation gas (nitrogen) flow rate were 2.5 kV, 15 V, 275 °C, and 650 L/h, respectively. LC separation was performed using a Waters HSS T3 column (2.1 × 150 mm, 1.8 µm particle size), with an injection volume of 3 µL at a flow rate of 0.25 mL/min (operated at 20 °C). The mobile phase consisted of solvent A (0.1% formic acid) and solvent B (0.1% acetonitrile). Features were separated by gradient conditions, with the initial condition 100% A and 0% B, followed by a linear gradient to 5% B over the first 1.10 min, 25% over 17 min, and, finally, 100% at 18 min. The column was subjected to 100% solvent B for an additional 3 min preceding a 5 min re-equilibration until the end of the total run time of 26 min. 

Compound detection and confirmation were performed as previously discussed by Magangana et al. [35]. This involved the use of certified reference standards of flavonoids and phenolics (Sigma-Aldrich, Darmstadt, Germany) to quantify individual compounds in pomegranate peel extracts. As well as the data acquisition, processing was conducted using MassLynx 4.1 software, according to our previous work Magangana et al. [35].

### 4.8. Determination of Minimum Inhibitory Concentration (MIC)

Determination of MIC was performed according to a method described by Eloff [76], with some modifications by Magangana et al. [45]. Briefly, the microorganisms used in this study consisted of two Gram-negative bacteria, *Escherichia coli* ATCC 11775 and *Klebsiella pneumoniae* ATCC 13883, and two Gram-positive bacteria, *Bacillus subtilis* ATCC 6051 and *Staphylococcus aureus* ATCC 12600. Streptomycin (100 µg/mL; Sigma Aldrich) was used as a positive control and bacteria-free broth, 70% (*v*/*v*) ethanol, and sterile distilled water were used as negative controls. The final concentration of peel extracts ranged from 19.53 to 2500 µg/mL, while streptomycin concentrations ranged from 0.20 to 25 µg/mL in the respective wells. The plates were left for 18 h at 37 °C for incubation purposes. After this period, 40 µL of *p*-iodonitrotetrazolium chloride (Sigma-Aldrich, Darmstadt, Germany) was added to the well plates to show bacterial growth. The results were expressed in terms of the MIC value, with this test performed in triplicate.

### 4.9. Statistical Analyses 

All data for phytochemical, antioxidant, and quantitative measurements of the biomarker phenolics were subjected to normality testing, preceding analysis of variance (ANOVA). Data that had a normal distribution were then subjected to a post-hoc analysis according to Duncan’s multiple range test to separate the means. Kruskal–Wallis analysis was used to separate the means as a post-hoc test for data that were not normally distributed. SAS Software (SAS Enterprise Guideline 7.1, Carrey, NC, USA) was used to calculate the statistical significance, with the results of all of the studied variables presented as mean ± S.E (*p* ≤ 0.05). All statistical analyses were conducted at the 95% confidence level.

For metabolomic analysis, the data were normalized using the sample median and transformed by log normalization, while data scaling was performed using the Pareto scaling algorithm prior to multivariate statistical analyses. A heat map, two-dimensional (2D) principal component analysis (PCA) score plot, variable importance in projection (VIP) plot, and orthogonal partial least squares discriminant analysis (OPLS-DA) were generated. In addition, a PCA biplot showing phenolic compound distribution and concentrations between the cultivars, namely ‘Wonderful’, ‘Acco’, and ‘Herskawitz’ pomegranate peel extracts, was also generated. All of the above plots were constructed using MetaboAnalyst 5.0 (www.metaboanalyst.ca; accessed on 20 November 2021). Pearson’s correlation matrix was constructed using GraphPad Prism software 4.03 (GraphPad Software, Inc., San Diego, CA, USA) to visualize relationships between phytochemical colourimetric analyses, antioxidant enzyme, and antibacterial assays plus biomarker phenolic metabolites in the data matrix.

## 5. Conclusions

This study has demonstrated for the first time that hot water blanching can promote high extraction of quality attributes such as phenolic content in various cultivars. Hot water blanching significantly reduced PPO and POD enzymatic activities in all three cultivars, namely ‘Acco’, ‘Herskawitz’, and ‘Wonderful’ peel extracts. By lowering enzymatic activity, the blanching effect contributed greatly to the high antioxidant and antibacterial activities reported in all blanched peel extracts, particularly those from blanched ‘Herskawitz’ peel extracts. Considerations for hot water blanching pre-treatment on a commercial scale should be made to process pomegranate peel for the formation and/or fortification of value-added products in the pharmaceutical, nutraceutical, and cosmeceutical industries. We recommend that a product manufacturer in the industry show interest in the up-regulation of key metabolites such as ellagitannins (galloyl-HHDP-DHHDP-hexoside, punicalin α and β, and α-punicalagin), phenolic acids (ellagic acid), and flavonoids (catechin, epicatechin) to blanch ‘Herskawitz’ peel extracts. While blanching of ‘Acco’ and ‘Wonderful’ pomegranate peel extracts may also increase the recovery of these key metabolites, they cannot be compared to the superior phytochemical profile obtained in blanched ‘Herskawitz’ peel extracts. This could result from inter-genetic cultivar variability, which may also be responsible for the thermal response of the cultivar during blanching. We recommend utilizing hot water blanching at 80 °C for 3 min as a pre-treatment step before processing the pomegranate peel. This study successfully preserved and recovered key metabolites from the peel extracts of three cultivars, namely ‘Acco’, ‘Herskawitz’, and ‘Wonderful’, by using hot water blanching at 80 °C for 3 min. We also recommend the application of ultrasonic extraction to those in the processing industry for faster results. Interestingly, although blanched ‘Wonderful’ peel extracts were reported with the highest extract yield, blanched ‘Herskawitz’ peel extracts were reported with the highest phytochemical and biological activity. Insights into the biochemical and genetic pathways using proteomic- and transcriptomic-based studies could provide an explanation. In addition, future studies should focus on the isolation of active compounds recovered in blanched ‘Herskawitz’ peel extracts and investigate more pharmacological activities (both in vitro and in vivo). We also recommend optimizing blanching temperature and time for ‘Herskawitz’ peel extracts using response surface methodology (RSM) and determining toxicity effects in the blanched peel extracts. In addition, a study of genetic variability between or within cultivars could provide significant information as to its potential for breeding purposes and novel drug discoveries. 

## Figures and Tables

**Table 1:** Extraction yield, bioactive compounds, enzyme activity, and antioxidant activity of ethanol (70% (*v*/*v*)) extracts from ‘Wonderful’, ‘Acco’, and ‘Herskawitz’ pomegranate peel cultivars at commercial harvest (ripe stage) blanched at 80 °C for 3 min.

Cultivar	Treatment	Extract Yield (%)	TPC	TTC	TFC	TAC	DPPH	FRAP	ABTS	PPO	POD
Wonderful	Unblanched	29.46 ± 0.75 ^b^	10.61 ± 0.15 ^d^	0.76 ± 0.02 ^f^	1.17 ± 0.00 ^c^	0.07 ± 0.00 ^f^	243.97 ± 2.43 ^d^	478.04 ± 73.98 ^e^	718.79 ± 2.43 ^f^	0.40 ± 0.00 ^b^	3.00 ± 0.00 ^b^
	Blanched	33.83 ± 1.42 ^a^	12.22 ± 0.08 ^c^	1.06 ± 0.06 ^e^	1.50 ± 0.00 ^b^	0.08 ± 0.00 ^e^	319.16 ± 4.20 ^c^	525.34 ± 15.77 ^d^	778.82 ± 2.43 ^d^	0.33 ± 0.03 ^c^	1.5 ± 0.00 ^c^
Acco	Unblanched	25.57 ± 0.68 ^cd^	13.71 ± 0.36 ^b^	1.27 ± 0.05 ^d^	1.00 ± 0.00 ^d^	0.09 ± 0.00 ^d^	429.53 ± 3.03 ^c^	703.63 ± 3.20 ^c^	758.21 ± 6.67 ^e^	0.68 ± 0.03 ^a^	4.25 ± 0.00 ^a^
	Blanched	28.96 ± 0.38 ^b^	15.47 ± 0.09 ^a^	1.70 ± 0.02 ^b^	1.17 ± 0.00 ^c^	0.11 ± 0.00 ^b^	585.37 ± 11.03 ^a^	772.15 ± 1.82 ^b^	869.18 ± 6.06 ^b^	0.28 ± 0.03 ^d^	3.00 ± 0.00 ^b^
Herskawitz	Unblanched	24.32 ± 0.55 ^d^	13.64 ± 0.27 ^b^	1.39 ± 0.02 ^c^	1.28 ± 0.06 ^c^	0.10 ± 0.00 ^c^	442.87 ± 7.28 ^b^	706.06 ± 4.85 ^c^	846.14 ± 3.03 ^c^	0.48 ± 0.08 ^b^	4.50 ± 0.00 ^a^
	Blanched	27.36 ± 0.60 ^bc^	15.74 ± 0.13 ^a^	1.92 ± 0.04 ^a^	1.70 ± 0.00 ^a^	0.15 ± 0.00 ^a^	567.78 ± 9.47 ^a^	800.05 ± 1.60 ^a^	915.27 ± 0.61 ^a^	0.25 ± 0.05 ^d^	3.13 ± 0.38 ^b^

Values are means ± SE of triplicate (*n* = 3) determinations. Superscripted letter(s) in each column indicate statistical significance (*p* ˂ 0.05) differences according to Duncan’s multiple range test. Extract yield (%) = percentage of gram of solvent extract per 100 g dried pomegranate peel powder, TPC = total phenolic content (mg GAE/g DM), TTC = total tannin content (mg GAE/g DM),TFC = total flavonoid content (CE per g DM), TAC = total anthocyanin content (mg C3GE/g DM), DPPH = 2,2 diphenyl-1-picryl hydrazyl assay (µmol Trolox/g DM), FRAP = ferric ion reducing antioxidant power assay (µmol Trolox/g DM), ABTS = 2,2-azino-bis(3-ethylbenzothiazoline-6-sulphonic acid assay (µmol Trolox/g DM), PPO = Polyphenol oxidase (U/g FW), POD = Peroxidase (U/g FW) unit per gram of fresh weight, GAE = gallic acid equivalent, CE = catechin equivalent, C3GE = Cyanidin-3-glucoside equivalent.

**Table 2:** Antibacterial activity (MIC, µg/mL) of ethanol (70% (*v*/*v*)) extracts from blanched (80 °C, 3 min) pomegranate peel extracts of cultivar ‘Wonderful’, ‘Acco’, and ‘Herskawitz’ at commercial harvest.

Cultivar	Treatment	Gram Negative		Gram Positive	
		*Escherichia coli*	*Klebsiella pneumoniae*	*Staphylococcus aureus*	*Bacillus subtilis*
**Wonderful**	**Unblanched**	310 ^b^	310 ^c^	310 ^b^	310 ^c^
	**Blanched**	**160 ^a^**	160 ^b^	**160 ^a^**	160 ^b^
**Acco**	**Unblanched**	310 ^b^	310 ^c^	310 ^b^	160 **^b^**
	**Blanched**	**160 ^a^**	160 ^b^	**160 ^a^**	**80 ^a^**
**Herskawitz**	**Unblanched**	310 ^b^	160 ^b^	310 ^b^	160 **^b^**
	**Blanched**	**160 ^a^**	**80 ^a^**	**160 ^a^**	**80 ^a^**
Significance level		<0.0001	<0.0001	<0.0001	<0.0001
**Streptomycin (µg/mL)**		1.6	1.6	0.8	1.6
**Solvent control (70% ethanol)**		-	-	-	-

Mean values in the same column followed by superscripted letter(s) represent statistical differences (*p* < 0.05) using Duncan’s multiple range test. All values in bold indicate those extracts that had the lowest minimum inhibitory concentration (MIC) values. - Denotes not inhibited.

**Table 3:** List of compounds tentatively identified in *P. granatum* L. (‘Wonderful, ‘Acco’, and ‘Herskawitz’) peel extracts showing retention times, detected [M − H]^−^ ion, elemental composition, MS^E^ fragments, and UV absorbance.

No.	Experimental *m/z* [M − H]^−^	Retention Time (min)	Elemental Formula	MS^E^ Fragments	UV (nm)	Tentative Identity	‘Wonderful’	‘Acco’	‘Herskawitz’	References
**1**	169.0146	4.72	C_7_H_5_O_5_	169.014, 125.025, 124.017	270; 259	Gallic acid *	√	√	√	Std
**2**	300.9969	14.24	C_14_H_5_O_8_	213.597, 137.983, 49.474	254; 364	Ellagic acid *	√	√	√	Std
**3**	463.0539	11.06	C_20_H_15_O_13_	463.053, 300.989, 165.021,114.6995	None	Ellagic acid hexoside	√	√	√	[35,36]
**4**	433.0307	13.59	C_19_H_13_O_12_	433.038, 303.7598, 300.995, 299.997, 201.556, 126.237	254; 361	Ellagic acid pentoside	√	√	√	[12,35,36]
**5**	447.0945	16.48	C_21_H_19_O_11_	447.074, 346.019, 327.124, 285.044, 284.025, 220.820, 143.011, 127.056, 51.022	230; 360	Ellagic acid rhamnoside	×	√	√	[11,36]
**6**	191.0198	2.52	C_5_H_7_O_7_	191.0198, 173.008, 111.008,87.008, 67.017	None	Citric acid *	√	√	√	Std
**7**	289.0733	8.31	C_15_H_13_O_6_	289.071, 245.082, 203.072, 109.028	None	(+)-Catechin *	√	√	√	Std
**8**	289.0733	10.19	C_15_H_13_O_6_	289.071, 245.082, 203.072, 109.028	None	(−)-Epicatechin *	√	√	√	Std
**9**	431.1906	11.07	C_20_H_20_O_10_	161.041, 153.091	253; 361	Apigenin-7-*O*-glucoside	√	√	√	[12,35]
**10**	593.1494	15.95	C_27_H_29_O_15_	593.142, 523.421, 440.063, 316.023, 300.998, 285.033, 211.911, 125.025, 101.031, 80.779	275; 360	Kaempferol-3-*O*-rutinoside	√	√	√	[35,37]
**11**	781.0506	4.99	C_34_H_21_O_22_	781.022, 779.000,783.055, 784.065	270; 259	Punicalin α and β *	√	√	√	Std
**12**	1083.0547	6.46	C_48_H_27_O_30_	1083.060, 781.035, 600.986, 541.027, 300.997	258; 378	α Punicalagin *	√	√	√	Std
**13**	1083.0558	7.69	C_48_H_27_O_30_	1083.060, 781.035, 600.986, 541.027, 300.997	258; 378	β Punicalagin *	√	√	√	Std
**14**	1083.0547	5.31	C_48_H_27_O_30_	1083.060, 781.035, 600.986, 541.027, 300.997	258; 378	Punicalagin isomer	√	√	√	Std
**15**	541.0341	6.44	-	541.027, 300.997	258; 378	Punicalagin derivative	√	√	√	[35,38]
**16**	951.0479	6.83	C_52_ H_23_O_19_	951.042, 907.087, 820.125, 783.062, 300.997, 275.021, 249.035, 102.241	255; 230	Galloyl-HHDP-DHHDP-hexoside	√	√	√	[12,35,38]
**17**	951.071	12.43	C_52_ H_23_O_19_	951.044, 933.079,915.9995, 763.081, 614.803, 464.044, 341.011, 302.006, 300.999, 273.002, 169.013, 1233.011	273	Galloyl-HHDP-DHHDP-hexoside (Granatin B)	√	√	√	[12,35,38]
**18**	799.0454	8.34	C_34_H_23_O_23_	799.0454, 800.0641, 802.0781	None	Granatin A	√	√	√	[10,11,35]
**19**	801.0732	8.60	C_41_H_21_O_18_	801.073, 781.3245		Digalloyl-HHDP-gluconic acid (punigluconin)	√	√	√	[10,35]
**20**	785.0775	9.37	C_34_H_25_O_22_	785.021, 781.795, 635.099, 483.122, 419.050, 345.086, 301.995, 300.9982, 275.032, 165.021, 125.132	264; 370	Digalloyl-HHDP-hexoside (Pedunculagin II)	√	√	√	[11,35,36,38]
**21**	633.0686	9.76	C_27_H_22_O_18_	633.075, 597.082, 464.051, 301.9998, 275.0219, 125.024	260;360	Galloyl-HHDP-hexoside	√	√	√	[35,38,39]
**22**	1085.0889	9.99	C_48_H_29_O_30_	933.081, 783.088, 633.0696, 540.536, 450.989, 301.996, 300.997, 275.014,	257; 370	Digalloyl-gallagyl-hexoside	√	√	√	[35,36,39]
**23**	783.0746	8.83	C_34_H_23_O_22_	783.567, 635.087, 541.022, 453.103, 392.036, 291.016, 203.0396	260; 370	Bis-HHDP-hexoside (pedunculagin I)	√	√	√	[35,36,38]
24	615.0828	13.01	C_23_H_19_O_20_ C_28_H_24_O_16_	615.056, 508.782, 324.158, 302.004, 301.000, 299.981, 190.991, 67.024	254; 230	Dehydro-galloyl-HHDP-hexoside	×	√	√	[12,39]
**25**	217.0461	1.58	C_12_H_9_O_4_	217.042, 191.0211, 173.006	None	Unknown a	√	√	√	[35]
**26**	353.0731	2.37	C_12_H_17_O_12_	353.0742, 191.0211, 173.0062	None	Unknown b	√	√	√	[35]
**27**	219.0497	6.43	C_8_H_11_O_7_	123.005, 111.009, 67.010	None	Unknown c	×	√	√	-
**28**	539.2146	12.81	C_26_H_35_O_12_	541.038, 492.1699, 405.014, 328.021, 302.001, 300.998, 222.005, 169.011, 52.256	254; 230	Unknown d	×	√	√	-
**29**	491.0756	15.27	C_22_H_19_O_13_	491.085, 357.434, 328.016, 312.996, 300.9995, 297.984, 211.149, 197.990, 55.027	None	Unknown e	√	√	√	[35]
**30**	187.1024	16.71	C_9_H_15_O_4_	100.092, 52.055	None	Unknown f	×	√	√	-

√ = present; × = not detected and * = confirmed using a pure chemical standard; - = unknown or no records in literature sources to our knowledge; literature sources and *standards were used to corroborate existing observations. MS^E^ fragments in bold refer to the base peak (the highest peak) [35].

**Table 4:** Pearson’s correlation matrix between chemical indices measured from blanched (80 °C for 3 min) ‘Acco’, ‘Herskawitz’, and ‘Wonderful’ pomegranate peel.

	POD	EY	GA	Epi	FRAP	DPPH	TTC	TPC	*B.s*	TAC	ABTS	Pα&β	*K.p*	*S.a*	*E.c*	TFC	βPC	PPO
POD	**1**																	
EY	**−0.98**	**1**																
GA	0.49	−0.50	**1**															
Epi	−0.05	−0.06	0.19	**1**														
FRAP	0.48	−0.59	0.55	0.70	**1**													
DPPH	0.28	−0.40	0.40	0.79	**0.97**	**1**												
TTC	0.21	−0.36	0.38	0.76	**0.95**	**0.97**	**1**											
TPC	0.25	−0.38	0.47	0.77	**0.97**	**0.99**	**0.98**	**1**										
*B.s*	−0.02	0.14	−0.47	−0.66	**−0.85**	**−0.91**	**−0.92**	**−0.94**	**1**									
TAC	0.16	−0.33	0.29	0.70	**0.85**	**0.86**	**0.95**	**0.88**	-0.78	**1**								
ABTS	0.11	−0.27	0.15	0.61	**0.84**	**0.89**	**0.95**	**0.89**	**−0.86**	**0.92**	**1**							
Pα&β	−0.19	0.02	0.23	0.72	0.71	0.78	**0.89**	**0.82**	**−0.84**	**0.93**	**0.87**	**1**						
*K.p*	0.25	−0.09	0.07	−0.36	−0.53	−0.63	−0.75	−0.66	0.75	−0.76	**−0.89**	**−0.84**	**1**					
*S.a*	0.70	−0.59	0.13	−0.59	−0.29	−0.48	−0.55	−0.51	0.67	−0.52	−0.59	−0.78	0.75	**1**				
*E.c*	0.70	−0.59	0.13	−0.59	−0.29	−0.48	−0.55	−0.51	0.67	−0.52	−0.59	−0.78	0.75	**1.00**	**1**			
TFC	−0.48	0.33	−0.18	0.15	0.14	0.22	0.42	0.28	−0.38	0.60	0.58	0.74	**−0.82**	−0.66	−0.66	**1**		
βPC	−0.61	0.47	−0.36	0.11	0.07	0.19	0.36	0.24	−0.39	0.49	0.57	0.67	**−0.86**	−0.75	−0.75	**0.96**	**1**	
PPO	0.65	−0.53	0.62	−0.43	−0.09	−0.29	−0.38	−0.27	0.32	−0.45	−0.55	−0.61	0.72	0.80	0.80	−0.71	**−0.83**	**1**

Values in bold are different from 0 with a significance level alpha = 0.05. Abbreviations: Extract yield (EY), total phenolic content (TPC), total tannin content (TTC), total flavonoid content (TFC), total anthocyanin content (TAC), 2,2-diphenyl-1-picryl hydrazyl (DPPH) free radical scavenging assay, ferric ion reducing antioxidant power (FRAP), polyphenol oxidase (PPO), peroxidase (POD), punicalin α&β (Pα&β), α-punicalagin (αPC), β-punicalagin (βPC), ellagic acid (EA), catechin (Cat), epicatechin (Epi), gallic acid (GA), *E. coli* (*E.c*), *K. pneumoniae* (*K.p*), *S. aureus* (*S.a*), *B. subtilis* (*B.s*).

## Data Availability

Not applicable.

## Supplementary Materials

The following supporting information can be downloaded at: https://www.mdpi.com/article/10.3390/molecules27092979/s1. Figure S1: Examples of chromatograms of ‘Acco’, ‘Herskawitz’, and ‘Wonderful’ pomegranate peel extracts obtained at ripe harvest maturity and blanched at 80 °C for 3 min. Compound numbers correspond to Table 3. Figure S2: Pearson’s correlation matrix heatmap between chemical indices measured from blanched (80 °C for 3 min) ‘Acco’, ‘Herskawitz’, and ‘Wonderful’ pomegranate peel extracts. Figure S3: (A) Orthogonal partial least squares discriminant analysis (OPLS-DA) for ‘Acco’ unblanched (purple colour) and ‘Acco’ blanched (green colour) peel samples. (B) S-plot of orthogonal partial least squares discriminant analysis of LC-MS spectra for ‘Acco’ unblanched and ‘Acco’ blanched peel samples showing the compounds that are important discriminants. (C) Variable importance in the projection (VIP) plot showing the 13 most statistically significant metabolites linked to the changes in ‘Acco’ unblanched and ‘Acco’ blanched peel extracts. Figure S4: (A) Orthogonal partial least squares discriminant analysis (OPLS-DA) for ‘Wonderful’ unblanched (purple colour) and ‘Wonderful’ blanched (green colour) peel samples. (B) S-plot of orthogonal partial least squares discriminant analysis of LC-MS spectra for ‘Wonderful’ unblanched and ‘Wonderful’ blanched peel samples showing the compounds that are important discriminants. (C) Variable importance in the projection (VIP) plot showing the 10 most statistically significant metabolites linked to the changes in ‘Wonderful’ unblanched and ‘Wonderful’ blanched peel extracts.
