# Supplementary material for: Antioxidant, Antimicrobial, and Metabolomic Characterization of Blanched Pomegranate Peel Extracts: Effect of Cultivar
Source: Molecules. 2022 May 6;27(9):2979. doi: 10.3390/molecules27092979 (PMC9101763; doi:10.3390/molecules27092979)
Supplement: Supplementary file 1 [file molecules-27-02979-s001.zip › molecules-1664934-supplementary.pdf]

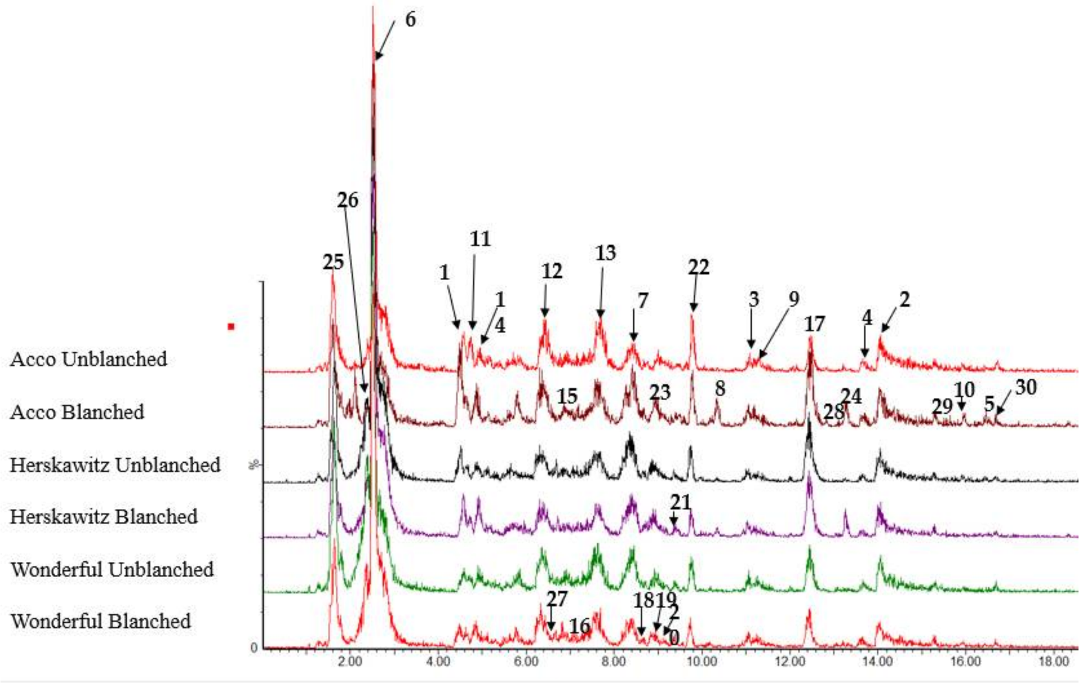

Figure S1. Examples of chromatograms of 'Acco', 'Herskawitz' and 'Wonderful' pomegranate peel extracts obtained at ripe harvest maturity, and blanched at 80 °C for 3 min. Compound numbers correspond to Table 4

.

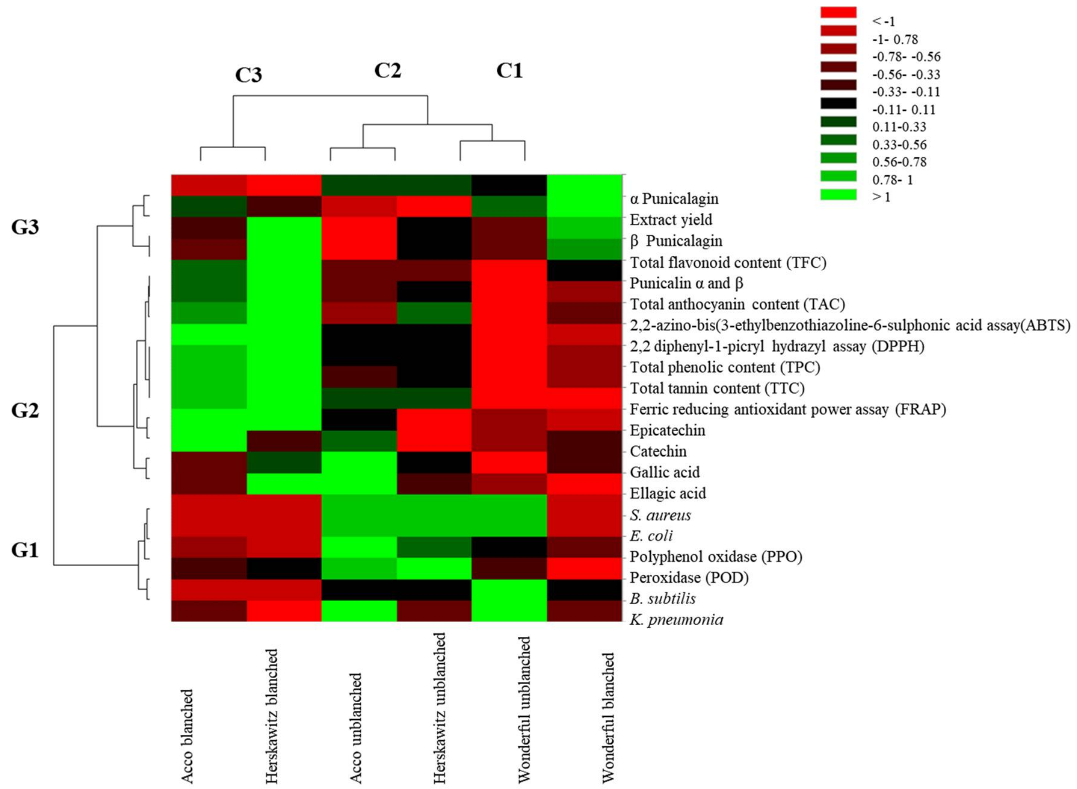

Figure S2. An example of "heatmap" showing a relationship of tested bioactives from different samples (cultivars). The correlation matrix is based on Pearson's coefficient scores showing correlation amongst tested parameters ($p < 0.05$). The dendrogram of samples (top) was divided into three parts (right (C1 or cluster 1), middle (C2 or cluster 2), and left cluster (C3 or cluster 3) based on the correlation between samples' cultivars and bioactives (also contains three groups (bottom group (G1 or group 1), (G2 or group2), (G3 or group 3), respectively. The green colour indicates positive correlations or high quantities and negative correlations or low quantities, are depicted in red and the intensity of the colour is proportional to the correlation coefficients.

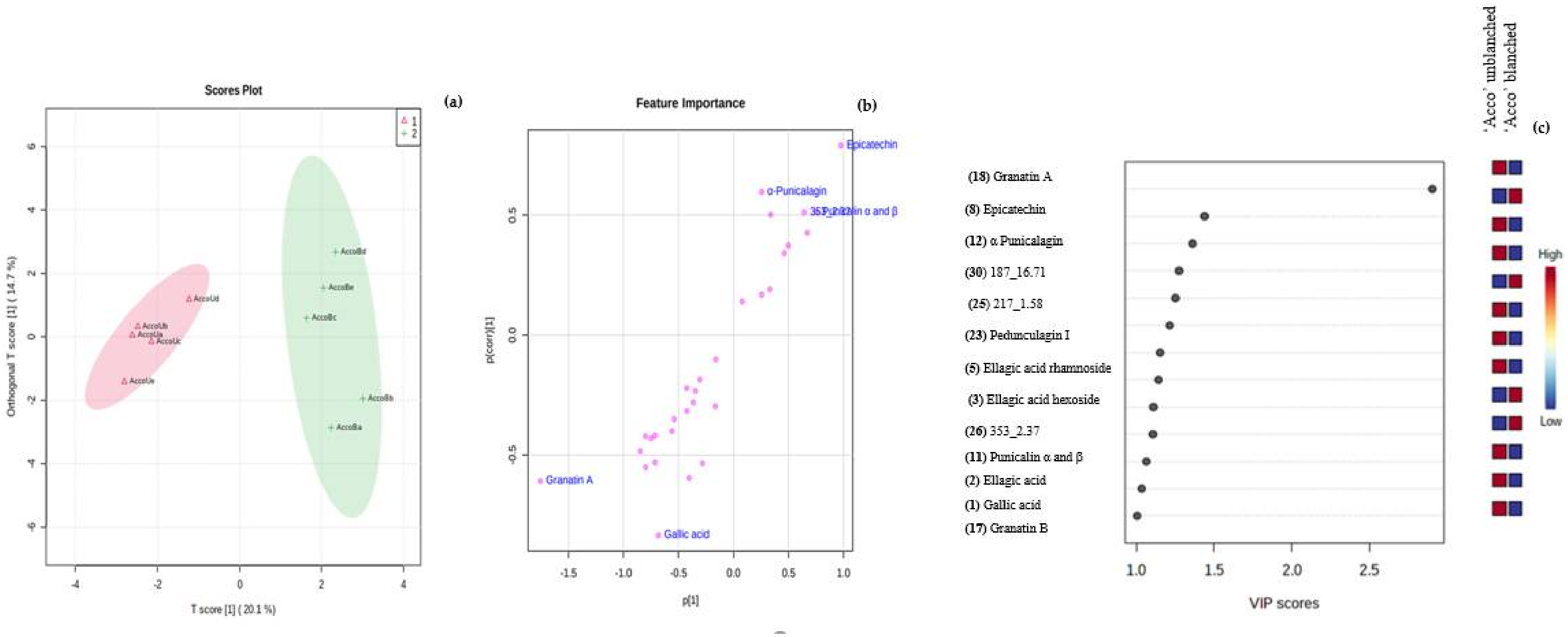

Figure S3. (**A**) Orthogonal partial least squares discriminant analysis (OPLS-DA) for 'Acco' unblanched (purple colour) and 'Acco' blanched (green colour) peel samples. **(B)** S-plot of orthogonal partial least squares discriminant analysis of LC-MS spectra for 'Acco' unblanched and 'Acco' blanched peel samples showing the compounds that are important discriminants. **(C)** Variable importance in the projection (VIP) plot showing the 13 most statistically significant metabolites linked to the changes in 'Acco' unblanched, and 'Acco' blanched peel extracts.

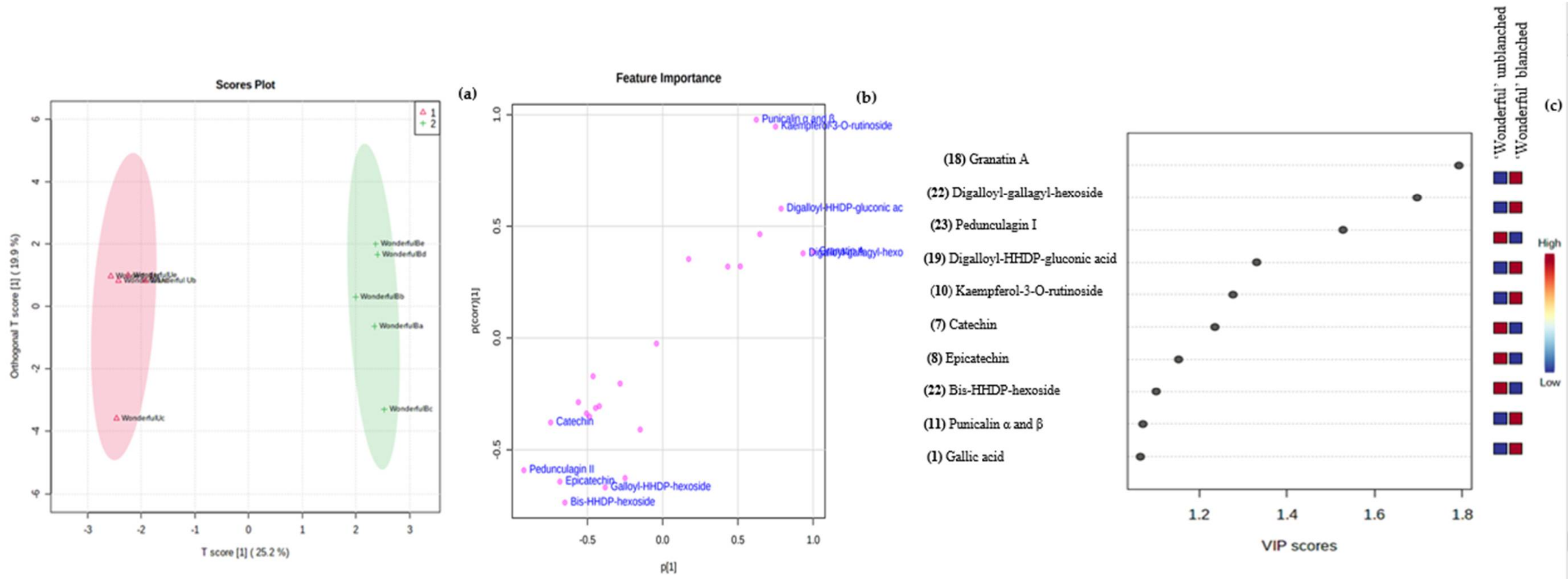

Figure S4. (**A**) Orthogonal partial least squares discriminant analysis (OPLS-DA) for 'Wonderful' unblanched (purple colour) and 'Wonderful' blanched (green colour) peel samples. **(B)** S-plot of orthogonal partial least squares discriminant analysis of LC-MS spectra for 'Wonderful' unblanched and 'Wonderful' blanched peel samples showing the compounds that are important discriminants. **(C)** Variable importance in the projection (VIP) plot showing the 10 most statistically significant metabolites linked to the changes in 'Wonderful' unblanched, and 'Wonderful' blanched peel extracts.

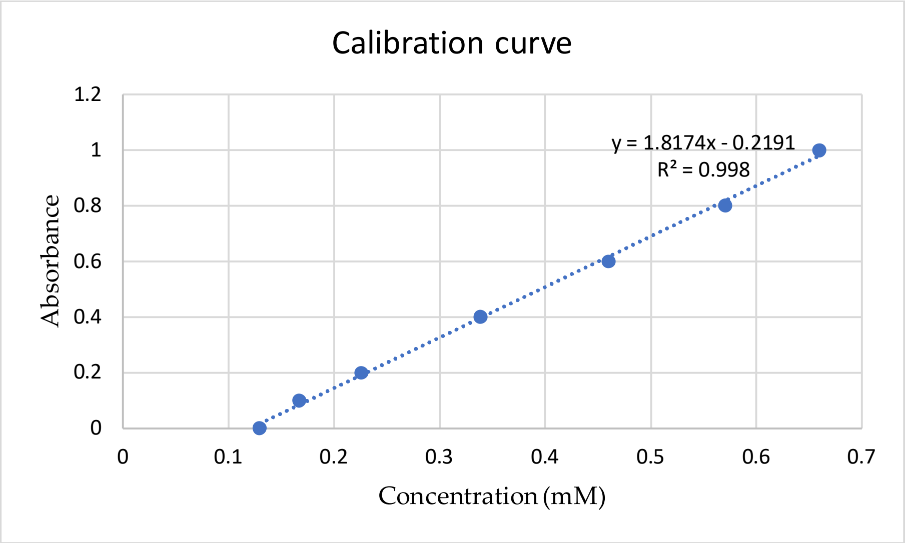

Figure S5. Calibration line used to calculate TEACs, for all the antioxidant tests (DPPH=2,2 diphenyl-1-picryl hydrazyl assay, FRAP=ferric reducing antioxidant power assay, ABTS=2,2-azino-bis(3-ethylbenzothiazoline-6-sulphonic acid assay).
